# Incidental Epiploic Appendagitis With Contralateral Anterior Cutaneous Nerve Entrapment Syndrome in an Adolescent

**DOI:** 10.14309/crj.0000000000002221

**Published:** 2026-07-09

**Authors:** Khayreddine Bouabida, Margarieta Okang

**Affiliations:** 1 Department of Pediatrics, UConn Health, University of Connecticut, Farmington, CT; 2 Department of Internal Medicine, University of Montreal Hospital Centre (CHUM), Montréal, Québec, Canada

**Keywords:** epiploic appendagitis, anterior cutaneous nerve entrapment syndrome (ACNES), abdominal wall pain, abdominal imaging, nerve block

## Abstract

Abdominal pain in adolescents often prompts imaging that may reveal incidental findings unrelated to the presenting symptoms, and anchoring on such findings can delay recognition of the true diagnosis. A 17-year-old girl with no significant past medical or surgical history presented to the emergency department with acute right lower quadrant pain of approximately 12 hours' duration. The pain was sharp, nonradiating, exacerbated by movement, and unrelated to meals. Contrast-enhanced computed tomography performed to evaluate for appendicitis revealed left lower quadrant epiploic appendagitis, anatomically discordant with her symptoms. The anatomic mismatch prompted a focused abdominal wall examination, which demonstrated a discrete right-sided trigger point with positive Carnett sign and altered cutaneous sensation, findings most consistent with anterior cutaneous nerve entrapment syndrome. Ultrasound-guided rectus sheath nerve block provided partial relief, and the patient was managed conservatively without surgery. Over subsequent months of multidisciplinary follow-up, her intermittent pain has been controlled with supportive care, with no new hospital admissions and no gynecologic pathology identified. This case highlights anterior cutaneous nerve entrapment syndrome as an underrecognized cause of focal abdominal pain in adolescents and illustrates the importance of anatomic correlation between imaging findings and clinical symptoms.

## INTRODUCTION

Abdominal pain is among the most common reasons for pediatric emergency department visits, yet establishing a definitive diagnosis remains challenging.^1,2^ Imaging frequently reveals incidental findings unrelated to the presenting symptoms, creating a risk of diagnostic anchoring.^3^

Epiploic appendagitis is a rare, self-limiting condition caused by torsion or venous thrombosis of an epiploic appendage, producing sharply localized pain at the site of inflammation, most commonly in the left lower quadrant.^4,5^ It typically resolves within 1 to 2 weeks with conservative management.^5^

Anterior cutaneous nerve entrapment syndrome (ACNES) results from entrapment of thoracoabdominal intercostal nerve branches (T7–T12) at the lateral border of the rectus abdominis, causing sharp localized pain that worsens with abdominal wall contraction.^6,7^ In 1 single-center pediatric cohort, ACNES was identified in approximately 1 in 8 adolescents with chronic abdominal pain, yet it remains widely underdiagnosed.^8^

## CASE REPORT

A 17-year-old girl with no significant past medical history, no previous abdominal surgeries, and no previous presentations for abdominal pain presented with right lower quadrant (RLQ) pain of approximately 12 hours' duration. The pain began suddenly at rest, was sharp, nonradiating, rated 9/10, exacerbated by movement, and unrelated to meals. She reported nausea and 2 episodes of nonbloody emesis, and denied fever, diarrhea, constipation, dysuria, hematuria, or menstrual irregularities. Her last menstrual period was 2 weeks prior.

Vital signs were stable and the patient was afebrile. Abdominal examination revealed RLQ tenderness with voluntary guarding but no rebound tenderness or rigidity. Laboratory studies including white blood cell count, comprehensive metabolic panel, lipase, C-reactive protein, and urinalysis were unremarkable. Urine pregnancy test was negative.

Abdominal and pelvic contrast-enhanced computed tomography showed a normal appendix, normal bilateral ovaries without cyst or torsion, no mesenteric lymphadenopathy, and no bowel wall thickening. However, a 2.9 × 2.7 cm ovoid area of fat stranding was identified in the left lower quadrant mesenteric fat anterior to the sigmoid colon (attenuation −60.7 HU), with the characteristic hyperattenuating ring sign consistent with primary epiploic appendagitis.^4,5^ This finding was anatomically discordant with her right-sided symptoms (Figure 1).

**Figure 1.**
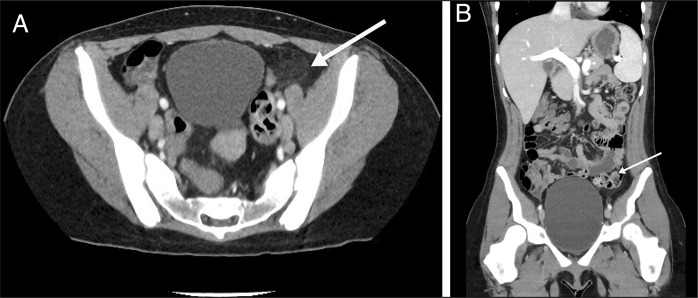
Contrast-enhanced computed tomography demonstrating primary epiploic appendagitis. (A) Axial image shows an ovoid area of fat stranding in the left lower quadrant (arrow), measuring 2.9 × 2.7 cm with attenuation of −60.7 HU, anterior to the sigmoid colon, consistent with inflamed fat typical of epiploic appendagitis. (B) Coronal image demonstrates the same lesion (arrow) lateral to the sigmoid colon. These findings are anatomically discordant with the patient's right lower quadrant pain.

The anatomic mismatch prompted a focused abdominal wall examination. A discrete trigger point was identified at the lateral border of the right rectus abdominis with pain intensifying during voluntary contraction (positive Carnett sign) and hypoesthesia in the T11–T12 dermatome. These findings met 3 of 4 diagnostic criteria for ACNES: sensory disturbances, positive Carnett sign, and trigger point tenderness, though the nerve block response did not reach the ≥50% threshold.^6^

The patient received nonsteroidal anti-inflammatory drugs for the epiploic appendagitis.^5,9^ An ultrasound-guided rectus sheath nerve block with 0.25% bupivacaine reduced pain from 9/10 to 6/10 (33% reduction), below the traditional ≥50% diagnostic threshold.^6,10^ A brief course of oral oxycodone (5 mg as needed) was administered during the initial 48-hour diagnostic phase consistent with the American Academy of Pediatrics 2024 guideline, alongside nonsteroidal anti-inflammatory drugs and acetaminophen as primary analgesics; opioids were discontinued before discharge with no outpatient prescription provided.^11^

At 2-week follow-up, symptoms had improved. Over subsequent months of multidisciplinary care with pain management, gastroenterology, nutrition, psychology, and physical therapy, her intermittent pain has been controlled with supportive care. She has had no new emergency department visits or hospital admissions. Gynecologic evaluation confirmed no reproductive pathology. Abdominal wall pain syndrome remains the most probable diagnosis.

## DISCUSSION

This case illustrates 2 complementary lessons: the importance of avoiding diagnostic anchoring when imaging findings are anatomically discordant with symptoms, and the recognition of ACNES as the most probable diagnosis that would have been missed had the team fixated on the left lower quadrant epiploic appendagitis.

Epiploic appendagitis produces localized somatic pain at the site of inflammation; contralateral pain has not been described.^5,7^ The differential diagnosis of acute RLQ pain in a 17-year-old girl includes appendicitis, ovarian pathology, mesenteric lymphadenitis, Crohn's disease, and abdominal wall pain.^1,2,12,13^ In this patient, computed tomography excluded appendicitis, ovarian pathology, lymphadenitis, and inflammatory bowel disease, while normal urinalysis excluded urinary tract infection. The trigger point, positive Carnett sign, and dermatomal hypoesthesia distinguished abdominal wall pain from visceral etiologies.^14,15^

The partial nerve block response (33% vs ≥50% threshold) may reflect suboptimal needle placement, the acute high-pain setting in which the block was performed, or the concurrent inflammatory state from the contralateral epiploic appendagitis.^6,10^ The diagnosis is therefore framed as probable rather than confirmed. Nevertheless, the overall clinical pattern — trigger point tenderness, positive Carnett sign, dermatomal hypoesthesia, absence of intra-abdominal pathology, and functional improvement over months — strongly supports ACNES as the most probable diagnosis.^6,14,15^

Diagnostic anchoring poses a significant risk when incidental imaging abnormalities are present.^3,16^ The clinical team's decision to perform a focused abdominal wall examination, prompted by the anatomic discordance, was the pivotal step that redirected the diagnostic pathway.^14,15^

From a longitudinal perspective, abdominal wall pain may recur and overlap with features of disorders of gut-brain interaction, particularly in patients with risk factors including female sex, triggering visceral events, and recurrent somatic symptoms.^6,13,17^ Structured multidisciplinary follow-up, as implemented here, represents a sound strategy for monitoring this trajectory.^6,17–19^

This case demonstrates that anatomic correlation between imaging findings and symptoms is essential for accurate diagnosis. By recognizing the discordance and performing a focused abdominal wall examination, the clinical team identified ACNES as the most probable diagnosis, avoiding unnecessary interventions. Both conditions were managed conservatively, and the patient has remained clinically stable over several months of multidisciplinary follow-up.

## DISCLOSURES

Author contributions: K. Bouabida conceptualized and designed the case report, acquired and analyzed the clinical data, drafted the original manuscript, critically revised it for important intellectual content, approved the final version to be published, and agrees to be accountable for all aspects of the work. M. Okang contributed to the acquisition and interpretation of clinical data, critically revised the manuscript for important intellectual content, approved the final version to be published, and agrees to be accountable for all aspects of the work. K. Bouabida is the article guarantor.

Acknowledgments: We thank the multidisciplinary medical team, including attending physicians, residents, nurses, and allied health staff, for their contributions to the patient's care. We also thank Dr Zempsky for his mentorship and thoughtful guidance in refining the manuscript. We acknowledge the radiology team for their expert interpretation of the imaging studies presented in this report.

Financial disclosure: None to report.

Informed consent was obtained for this case report.

## ABBREVIATIONS:

AAP: American Academy of Pediatrics
ACG: American College of Gastroenterology
ACNES: anterior cutaneous nerve entrapment syndrome
ACR: American College of Radiology
CHUM: Centre hospitalier de l'Université de Montréal (University of Montreal Hospital Centre)
CT: computed tomography
HU: hounsfield units
NSAIDs: nonsteroidal anti-inflammatory drugs
RLQ: right lower quadrant
T7–T12: thoracic spinal nerve levels 7 through 12
UConn: University of Connecticut
